# POLICY SERIES: POSITIVE EXPERIENCES OF AGING AND HEALTH: COMPLEMENTARY EVIDENCE FROM TWO STUDIES

**DOI:** 10.1093/geroni/igad104.0287

**Published:** 2023-12-21

**Authors:** Julie Ober Allen, Valerie Moïse, C J White, Erica Solway

**Affiliations:** University of Oklahoma, Norman, Oklahoma, United States; Stephenson Cancer Center at OU Medicine, Oklahoma City, Oklahoma, United States; University of Oklahoma, Norman, Oklahoma, United States; University of Michigan, Ann Arbor, Michigan, United States

## Abstract

Social constructions of aging and associated treatment of older adults are predominantly negative (i.e., ageism) and believed to be harmful for health. Societal beliefs, expectations, and practices linked to old age may also be associated with benefits, such wisdom, respect, greater self-acceptance, more focus on family and chosen activities, and eligibility for age-specific privileges. Positive experiences of aging such as these have not been extensively researched. This study investigated the prevalence of positive experiences of aging and their relationships with health using data from two complementary studies: 5 items focused on positive experiences of aging in a nationally representative sample (2019 National Poll on Healthy Aging (NPHA); Mage=62.6, 54.2% female); and 23 items in a smaller exploratory study (2021-2022 Experiences of Aging in Society (EOA); Mage=71.1, 81.8% female). Associations with health were assessed using multivariable regression. Report of positive experiences of aging was common (NPHA: >65% for all items; EOA: >80% for 20). Most frequently endorsed were being treated with respect, value for one’s opinions, wanting to be a good example to younger people, greater acceptance of not work full time and spending time with family (all EOA), and feeling more comfortable with oneself (NPHA and EOA). Positive experiences of aging were associated with better health (NPHA: fewer chronic health conditions (p=.023) and reduced risk of low self-rated physical health, mental health, and depressive symptoms (p-values <.001); EOA: fewer chronic health conditions (p=.004). Increasing positive experiences of aging may be a promising strategy for promoting older adult health and wellbeing.

